# Next-generation sequencing yields the complete mitogenome of the stored nut moth, *Paralipsa gularis* Zeller (Lepidoptera: Pyralidae)

**DOI:** 10.1080/23802359.2021.1915204

**Published:** 2021-08-16

**Authors:** Ping Guo, Hong Yu, Jin Xu, Yong-He Li

**Affiliations:** a Key Laboratory for Forest Resources Conservation and Utilization in the Southwest Mountains of China, Ministry of Education, Southwest Forestry University, Kunming, PR China; b Yunnan Academy of Biodiversity, Southwest Forestry University, Kunming, PRChina

**Keywords:** Mitochondrial genome, *Paralipsa gularis*, Pyralidae, stored product pest

## Abstract

The stored nut moth, *Paralipsa gularis* Zeller 1877 (Lepidoptera: Pyralidae), is a pest of stored products. In this study, the whole mitogenome of *P. gularis* was identified for the first time by using the next-generation sequencing (NGS) systems. The entire genome is 15,280 bp in length (ACCN: MW135332) consisting of 13 protein-coding genes (PCGs), two ribosomal *RNA* genes, 22 transfer *RNA* genes, and an A + T-rich region. Phylogenetic analysis using 13 PCGs of 20 species derived from six moth superfamilies showed that Pyralidae moths are monophyletic. This study can provide essential DNA molecular data for further phylogenetic and evolutionary analysis for Pyralidae family of Lepidoptera order.

The stored nut moth *Paralipsa gularis* Zeller 1877 (synonym: *Aphomia gularis*) is a resident of south-east Asia (Wang 1980; Tai et al. 2018), which spread to India, North Korea, Japan, Northern Europe, and North America with the food trade (Trematerra 1987; Kageyama et al. 2010; Hong et al. 2012). This is a sexually dimorphic species, with the males being smaller and more delicately marked than the females. The larvae feed on stored seeds and nuts, as well as dried fruit and mixed dried food (Wang 1980; Tai et al. 2018).

In this study, *P. gularis* was collected in 2019 in Jiangchun of Yunnan province, China and the specimen (voucher no. M2019-0036) was deposited in the Insect Systematics and Diversity Lab (contact person: Hong Yu; email: yuhong1652@126.com) at Southwest Forestry University, Kunming, China. Genomic DNA was extracted from the whole body of a single *P. gularis* larva with phenol-chloroform, precipitated with isopropanol and sodium acetate (300 mM), and dissolved in Tris-EDTA (TE) buffer. The isolated DNA was sheared to 500-bp fragments in a Covaris (KBiosciences, Herts, UK) ultrasonicator device for preparing the next-generation sequencing (NGS) library using the paired-end NEBNext Ultra DNA Library Prep Kit for Illumina (Illumina, San Diego, CA). Sequencing using NovaSeq (Illumina, San Diego, CA) generated 16,175,508 clean reads. Clean reads were de novo assembly by using commercial software (Geneious version 8, Auckland, New Zealand) to produce a single, circular form of complete mitogenome with about an average 167× coverage. The rRNA, tRNA, and protein-coding genes of *P. gularis* mitogenome were predicted by using MITOS (Bernt et al. 2013), DOGMA (Wyman et al. 2004), and ARWEN (Laslett and Canback 2008) software and manually inspected.

The complete mitogenome of *P. gularis* was 15,280 bp in size and its overall base composition is 39.2% for A, 40.3% for T, 7.8% for G, and 12.7% for C, and have GC content of 20.5%. It contains 13 protein-coding genes (PCGs), 22 transfer *RNA* genes (*tRNA*s), two ribosomal *RNA* genes (*rRNA*s), and a major non-coding adenine (A) + thymine (T)-rich region. The A + T-rich region is 320 bp long and located between 12S rRNA and tRNA-Met. The A + T content is a parameter which was usually used in the investigation of the nucleotide-compositional behavior of mitogenome (Hassanin et al. 2005; Song et al. 2016). All of the PCGs have ATN as the start codon except for cox1, which starts with CGA. Ten PCGs have the common stop codon TAA, while cox2, nad1, and nad4 have the incomplete stop codon T.

Nucleotides sequences of 13 PCGs were used to understand the phylogenetic relationship of *P. gularis* with other moths by using MEGA version 6.0 software (Tamura et al. 2013) with maximum-likelihood (ML) method (with 1000 bootstrap replicates and the General Time Reversible model). Phylogenetic analysis revealed that insects from the same superfamily were clustered together (Figure 1). *Ephestia kuehniella*, *Plodia interpunctella*, *Lista haraldusalis*, *Corcyra cephalonica*, and *P. gularis* clusters with a 100% bootstrap value with the monophyletic Pyralidae family.

**Figure 1.**
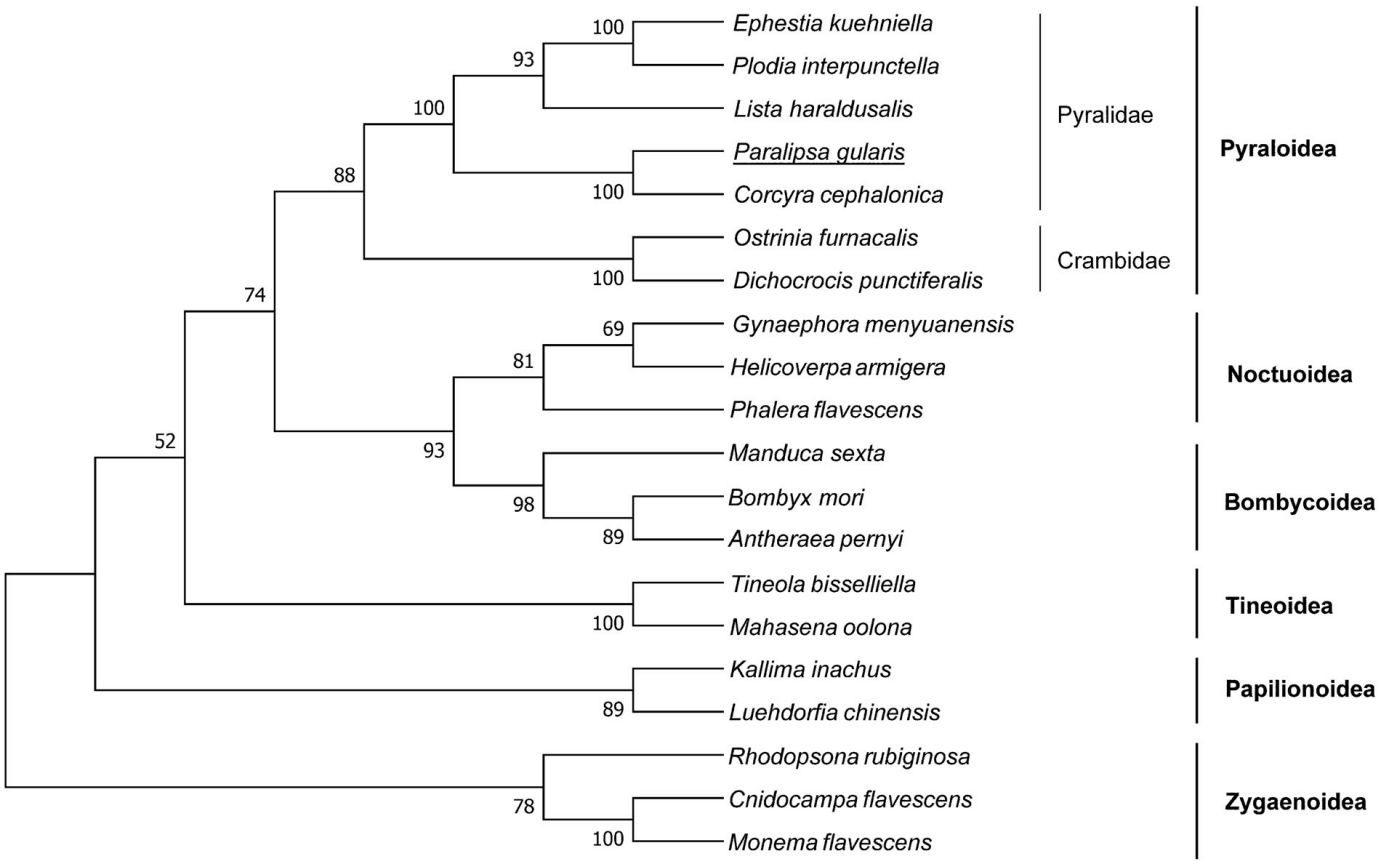
The maximum-likelihood (ML) phylogenetic tree of *Paralipsa gularis* and other moths. The GenBank accession numbers used for tree constructed are as follows: *Ephestia kuehniella* (NC_022476), *Plodia interpunctella* (KT207942), *Lista haraldusalis* (KF709449), *Corcyra cephalonica* (NC_016866), *Ostrinia furnacalis* (AF467260), *Dichocrocis punctiferalis* (NC_021389), *Gynaephora menyuanensis* (KC185412), *Helicoverpa armigera* (GU188273), *Phalera flavescens* (JF440342), *Manduca sexta* (EU286785), *Bombyx mori* (AF149768), *Antheraea pernyi* (HQ264055), *Tineola bisselliella* (KJ508045), *Mahasena oolona* (KY856825), *Kallima inachus* (JN857943), *Luehdorfia chinensis* (KM453727), *Rhodopsona rubiginosa* (KM244668), *Cnidocampa flavescens* (KY628213), and *Monema flavescens* (KU946971).

In conclusion, the whole mitogenome of *P. gularis* was identified for the first time in this study and can provide essential DNA molecular data for further phylogenetic and evolutionary analysis for Pyralidae family of Lepidoptera order.

## Data Availability

Mitogenome data supporting this study are openly available in GenBank at: https://www.ncbi.nlm.nih.gov/nuccore/MW135332. Associated BioProject, SRA, and BioSample accession numbers are https://www.ncbi.nlm.nih.gov/bioproject/ PRJNA675975, https://www.ncbi.nlm.nih.gov/sra/SRR13038347, and SAMN16745531, respectively.
